# Estratégia Chave para Prever Doenças Cardiovasculares: Uma Combinação de Índices Antropométricos

**DOI:** 10.36660/abc.20210716

**Published:** 2021-10-06

**Authors:** José Bruno Nunes Ferreira Silva

**Affiliations:** 1 Universidade Federal do Tocantins PalmasTO Brasil Curso de Medicina - Universidade Federal do Tocantins, Palmas, TO - Brasil

**Keywords:** Antropometria, Doenças Cardiovasculares, Obesidade, Pandemias, Fatores de Risco

A pandemia de obesidade é um dos problemas de saúde mais urgentes do século XXI. Prevalente mesmo em países desenvolvidos e em desenvolvimento, essa doença tem sido relacionada a eventos cardiovasculares, dislipidemia, diabetes tipo 2, distúrbios do sono e câncer. Recentemente, foi considerada um fator de risco para gravidade na doença causada pelo novo coronavírus-2019 (COVID-19).^1 , 2^

No Brasil, a Pesquisa Nacional de Saúde, em parceria com o Ministério da Saúde, constatou que a obesidade tem aumentado gradativamente nos últimos 17 anos em mulheres (14,5% a 30,2%) e homens (9,6% a 22,8%) adultos. Sua prevalência mais do que dobrou entre 2003 e 2019.^3^ Um esforço colaborativo entre o governo, funcionários de saúde pública e equipe médica é necessário para lidar com o problema complexo e multifatorial da obesidade.

Na prática clínica diária, a obesidade é avaliada por meio da medida do peso corporal e da gordura excessiva. Além do índice de massa corporal (IMC), índices antropométricos independentes, incluindo a circunferência da cintura (CC), relação cintura-quadril (RCQ) e índice de adiposidade corporal (IAC), são meios acessíveis para avaliar a obesidade abdominal. Apesar da disponibilidade e segurança na obtenção desses parâmetros, os índices adequados para prever o desenvolvimento de doenças cardiovasculares (DCV) em diferentes idades, sexos e etnias permanecem desconhecidos.^4^

Vários estudos brasileiros obtiveram resultados conflitantes ao avaliar as medidas antropométricas e o risco de desenvolvimento de DCV em adultos. Barroso et al. descobriram que a obesidade central, mas não o IMC, foi um preditor de DCV em mulheres adultas.^5^ A CC foi considerada a ferramenta alternativa de triagem mais adequada para identificar mulheres quilombolas com risco mais significativo de DCV.^6^ Outro estudo relatou que o IMC, a CC e a RCQ foram preditores viáveis de DCV em homens, enquanto o IMC foi o preditor mais confiável para mulheres.^7^ Hachbardt et al.^8^ recentemente demonstraram que o IMC estava associado à CC, relacionado ao risco de DCV, mas não à RCQ.^8^ Ao resolver os resultados conflitantes desses estudos, o indicador antropométrico padrão-ouro de adiposidade para predizer DCV pode ser estabelecido. Esses estudos foram realizados em diferentes períodos de tempo e seus tamanhos de amostra não eram representativos de toda a população do país.

O Estudo Longitudinal de Saúde do Adulto (ELSA-Brasil) relatou a associação entre indicadores antropométricos de obesidade e DCV em uma grande amostra de mulheres e homens.^9^ O parâmetro da circunferência do pescoço e o IAC foram propostos como preditores de um risco de 10 anos para o desenvolvimento de doença coronariana.^10 , 11^

Nesta edição dos Arquivos Brasileiros de Cardiologia, a equipe do ELSA-Brasil propôs um novo método de previsão de DCV baseado na combinação dos parâmetros antropométricos. O primeiro mérito do estudo foi que Almeida et al.^12^ foram o primeiro grupo a investigar a capacidade preditiva de parâmetros antropométricos individuais ou combinados para risco coronariano em 15.092 brasileiros.^12^ Além da análise individual dos parâmetros de obesidade, os autores criaram quatro combinações (IAC mais CC, IMC mais CC, IAC mais RCQ e IMC mais RCQ) para testar suas hipóteses. O segundo ponto positivo do estudo foi associar um parâmetro de obesidade geral a um indicador de obesidade central. Para caracterizar um risco aumentado para eventos cardiovasculares, os indivíduos foram classificados nos seguintes grupos: VHR20% - para aqueles com 20% ou mais de risco de doença arterial coronariana em 10 anos; e HR10%, para indivíduos com 10-20% de risco de doença arterial coronariana em 10 anos.

Abaixo estão as principais conclusões do estudo: Para parâmetros individuais, a RCQ foi o parâmetro mais confiável para VHR20% em ambos os sexos. Ao analisar as combinações, RCQ mais IAC foi o preditor mais confiável para homens, enquanto RCQ mais IMC foi o preditor mais confiável para mulheres com VHR 20%. Além disso, indivíduos com HR10% tiveram um risco maior de doença coronariana em 10 anos quando um IAC ou IMC aumentado foi combinado com uma CC ou RCQ mais elevada.^12^ Em resumo, as combinações de pelo menos um parâmetro de obesidade geral e um parâmetro de obesidade central foram os principais preditores de desenvolvimento de risco coronariano em 10 anos.

Esses dados destacaram a importância de incluir medidas agrupadas para analisar perfis de DCV. As informações obtidas neste estudo são essenciais para a revisão e atualização das diretrizes clínicas e das políticas públicas de saúde. A população brasileira é heterogênea em termos de condições sociais, econômicas e de saúde.^12^ Assim, análises futuras devem incluir um tamanho de amostra representativo para melhor compreender esta doença e seu manejo. Além disso, a pesquisa de Almeida et al.^12^ pode abrir caminhos para novos estudos que identifiquem os marcadores ideais em outros grupos, como a população jovem brasileira, comparando a associação de parâmetros antropométricos combinados no desenvolvimento do risco de DCV.

É hora de agir para proteger nossos corações.
