# Supplementary material for: Estratégia Chave para Prever Doenças Cardiovasculares: Uma Combinação de Índices Antropométricos
Source: Arq Bras Cardiol. 2021 Oct 6;117(4):713–4. [Article in Portuguese] doi: 10.36660/abc.20210716 (PMC8528361; doi:10.36660/abc.20210716)
Supplement: Translation

# Translation: Key Strategy to Predict Cardiovascular Diseases: A Combination of Anthropometric Indices

**Authors:** José Bruno Nunes Ferreira Silva

**Affiliations:** 1 Brazil Curso de Medicina - Universidade Federal do Tocantins, Palmas, TO - Brazil

**Keywords:** Anthropometry, Cardiovascular Diseases, Obesity, Pandemics, Risk Factors

The obesity pandemic is one of the most pressing health concerns of the 21^st^century. Prevalent even among developed and developing countries, this disease has been related to cardiovascular events, dyslipidemia, type 2 diabetes, sleep disorders, and cancer. It has recently been considered a risk factor for critical illness in coronavirus disease-2019 (COVID-19) pandemic.^1 , 2^

In Brazil, the National Health Survey , in partnership with the Ministry of Health, found that obesity has gradually increased over the last 17 years in adult women (14.5% to 30.2%) and men (9.6% to 22.8%). Its prevalence has more than doubled between 2003 and 2019.^3^ A collaborative effort between the government, public health officers, and medical staff is needed to manage the complex and multifactorial obesity issue.

In everyday clinical practice, obesity is assessed using body weight and excessive fat measurement. In addition to the body mass index (BMI), independent anthropometric indices, including the waist circumference (WC), waist-to-hip ratio (WHR), and body adiposity index (BAI), are accessible means for evaluating abdominal obesity. Despite the availability and safety when obtaining these parameters, the appropriate indices for predicting the development of cardiovascular disease (CVD) across different ages, genders, and ethnicity, remain unknown.^4^

Several Brazilian studies have obtained conflicting results when evaluating the anthropometric measures and risk of CVD development in adults. Barroso et al. found that central obesity, but not BMI, was a predictor of CVD in adult women.^5^ WC was considered the most appropriate alternative screening tool to identify Quilombola women with the most significant risk for CVD.^6^ Another study reported that the BMI, WC, and WHR were viable predictors of CVD in men, while BMI was the most reliable predictor for women.^7^ Hachbardt et al.^8^ recently showed that BMI was associated with WC, related to CVD risk, but not WHR.^8^ By resolving the conflicting results of these studies, the gold standard anthropometric indicator of adiposity for predicting CVD can be established. These studies were conducted in different time periods, and their sample sizes were not representative of the country’s entire population.

The Brazilian Longitudinal Study of Adult Health (ELSA-Brazil) has reported the association between anthropometric indicators of obesity and CVD in a large sample of women and men.^9^ The neck circumference parameter and BAI index have been proposed as predictors of a 10-year risk for developing coronary disease.^10 , 11^

In this issue of the *Arquivos Brasileiros de Cardiologia* , the ELSA-Brazil team has proposed a novel CVD prediction method based on the combination of the anthropometric parameters. The first merit of the study was that Almeida et al.^12^ were the first group to investigate the predictive ability of individual or combined anthropometric parameters for coronary risk in 15,092 Brazilian individuals.^12^ In addition to the individual analysis of obesity parameters, the authors created four combinations (BAI plus WC, BMI plus WC, BAI plus WHR, and BMI plus WHR) to test their hypotheses. The second positive point of the study was to associate one overall obesity parameter with one central obesity indicator. To characterize an increased risk for cardiovascular events, individuals were classified in the following groups: VHR20% - for those with 20% or more of a 10-year coronary artery disease risk; and HR10%, for subjects with 10-20% of a 10-year coronary artery disease risk.

Below are the main findings of the study: For individual parameters, WHR was the most reliable parameter for VHR20% in both sexes. When analyzing combinations, WHR plus BAI was the most reliable predictor for men, while WHR plus BMI was the most reliable predictor for women with VHR20%. In addition, individuals with HR10% had a higher 10-year coronary disease risk when an increased BAI or BMI was combined with a higher WC or WHR.^12^ In summary, the combinations of at least one overall obesity parameter and one central obesity parameter were the main predictors of coronary risk development in 10 years.

These data highlighted the importance of including grouped measures for analyzing CVD profiles. The information obtained in this study is essential for reviewing and updating the clinical guidelines and public health policies. The Brazilian population is heterogenous in terms of social, economic, and health conditions.^12^ . Thus, future analyses should include a representative sample size to better understand this disease and its management. Also, the research of Almeida et al.^12^ can open up ways for new studies that will identify the ideal markers in other groups, as the Brazilian youth population, comparing the association of combined anthropometric parameters on CVD risk development.

It is time to act to protect our hearts.
